# Tumor cell enrichment by tissue suspension improves sensitivity to copy number variation in diffuse gastric cancer with low tumor content

**DOI:** 10.1038/s41598-024-64541-3

**Published:** 2024-06-13

**Authors:** Keiichi Hatakeyama, Koji Muramatsu, Takeshi Nagashima, Hiroyuki Ichida, Yuichi Kawanishi, Ryutaro Fukumura, Keiichi Ohshima, Yuji Shimoda, Sumiko Ohnami, Shumpei Ohnami, Koji Maruyama, Akane Naruoka, Hirotsugu Kenmotsu, Kenichi Urakami, Yasuto Akiyama, Takashi Sugino, Ken Yamaguchi

**Affiliations:** 1 Cancer Multiomics Division, Shizuoka Cancer Center Research Institute, Sunto-gun, Shizuoka, 411-8777 Japan; 2 Division of Pathology, Shizuoka Cancer Center, Sunto-gun, Shizuoka, 411-8777 Japan; 3 Cancer Diagnostics Research Division, Shizuoka Cancer Center Research Institute, Sunto-gun, Shizuoka, 411-8777 Japan; 4 SRL Inc., Shinjuku-ku, Tokyo, 163-0409 Japan; 5 SRL and Shizuoka Cancer Center Collaborative Laboratories Inc., Sunto-gun, Shizuoka, 411-8777 Japan; 6 Medical Genetics Division, Shizuoka Cancer Center Research Institute, Sunto-gun, Shizuoka, 411-8777 Japan; 7 Experimental Animal Facility, Shizuoka Cancer Center Research Institute, Sunto-gun, Shizuoka, 411-8777 Japan; 8 Drug Discovery and Development Division, Shizuoka Cancer Center Research Institute, Sunto-gun, Shizuoka, 411-8777 Japan; 9 Division of Thoracic Oncology, Shizuoka Cancer Center, Sunto-gun, Shizuoka, 411-8777 Japan; 10 Immunotheraphy Division, Shizuoka Cancer Center Research Institute, Sunto-gun, Shizuoka, 411-8777 Japan; 11 Shizuoka Cancer Center, Sunto-gun, Shizuoka, 411-8777 Japan

**Keywords:** Cancer genomics, Gastrointestinal cancer, Next-generation sequencing

## Abstract

The detection of copy number variations (CNVs) and somatic mutations in cancer is important for the selection of specific drugs for patients with cancer. In cancers with sporadic tumor cells, low tumor content prevents the accurate detection of somatic alterations using targeted sequencing. To efficiently identify CNVs, we performed tumor cell enrichment using tissue suspensions of formalin-fixed paraffin-embedded (FFPE) tissue sections with low tumor cell content. Tumor-enriched and residual fractions were separated from FFPE tissue suspensions of intestinal and diffuse-type gastric cancers containing sporadic tumor cells, and targeted sequencing was performed on 225 cancer-related genes. Sequencing of a targeted panel of cancer-related genes using tumor-enriched fractions increased the number of detectable CNVs and the copy number of amplified genes. Furthermore, CNV analysis using the normal cell-enriched residual fraction as a reference for CNV scoring allowed targeted sequencing to detect CNV characteristics of diffuse-type gastric cancer with low tumor content. Our approach improves the CNV detection rate in targeted sequencing with tumor enrichment and the accuracy of CNV detection in archival samples without paired blood.

## Introduction

Cancer-related gene testing panel using next-generation sequencing (NGS) has expanded opportunities for drug selection in cancer treatment^1^. Several drugs have been developed for solid tumors with copy number variations (CNV) in cancer-related genes (e.g., *ERBB2*, *FGFR1/2*, *MET*, *MDM2*), and panel testing is being increasingly used to recruit patients for clinical trials. In addition, understanding CNVs in cancer-related genes is expected to lead to the discovery of candidate genes that may serve as new drug targets^2,3^. Recently, a relationship between therapeutic resistance, drug sensitivity, and copy number alterations has also been revealed^4–6^. Therefore, accurate evaluation of CNVs in targeted sequencing, where the number of genes is limited, has become important for therapeutic selection.

NGS is influenced by tumor content and often underestimates somatic alterations, especially in tumors with low tumor content^7^. Furthermore, large-scale analyses have revealed that focal copy number alterations are more sensitive to tumor content than to mutation detection^8,9^. Macrodissection, which removes tumor-rich regions to improve the detection of somatic alterations in tumors with low tumor content, has been performed in panel testing using formalin-fixed paraffin-embedded (FFPE) tissue sections^10,11^. However, this enrichment approach is frequently unsuitable for cancers containing sporadic tumor cells, such as diffuse-type gastric cancer and lobular breast cancer. Laser capture microdissection can also harvest tumor cells at the single-cell level but requires special equipment and expertise to obtain a substantial amount of DNA needed for NGS^12^. We previously reported a simple, semi-automated method for enriching diffuse-type tumor cells to improve somatic mutation detection^13^. This method focused on extracting DNA available for NGS after tumor enrichment and was performed on a small number of samples. The analysis pipeline was constructed solely for mutation detection.

In the present study, to efficiently detect CNV in diffuse-type gastric cancers with low tumor content, CNV analysis was performed after tumor cell enrichment of FFPE tissue sections. Copy number alterations were analyzed in a panel loaded with cancer-related genes using NGS to investigate the effects of tumor cell enrichment on CNV detection. In addition, CNVs were reanalyzed using the normal cell-rich residue fraction isolated via tumor enrichment as a normal equivalent reference to improve the accuracy of CNV evaluation without a germline/normal reference. Our approach to tumor cell enrichment and isolation allows for improved CNV detection rates in targeted sequencing and accurate identification of genes with CNV.

## Results

### Tumor cell enrichment

**Figure 1:**
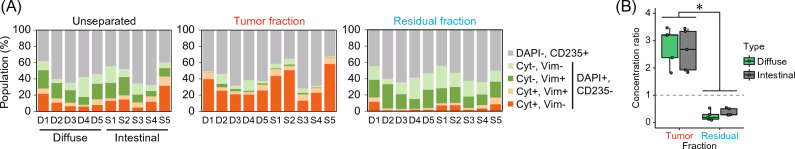
Estimation of tumor cell population in formalin-fixed paraffin-embedded (FFPE) tissue sections using flow cytometry. (**A**) The FFPE tissue sections were suspended and separated using magnetic-activated cell sorting (MACS) using anti-cytokeratin microbeads. These fractions were stained with anti-cytokeratin (Cyt) and vimentin (Vim) antibodies. DAPI and CD235 staining were performed simultaneously to distinguish nuclei and erythrocytes. Suspensions enriched with the microbeads were defined as the tumor fraction, and samples that could not be captured by these beads were discriminated as the residual fraction. Suspensions that were not subjected to MACS were defined as unseparated. (**B**) This ratio is defined as the tumor enriched population (Cyt+, Vim−) in each fraction divided by that in the unseparated sample. Asterisk represents significance at *p* < 0.01.

FFPE tissue sections of diffuse-type and intestinal gastric cancer were suspended for tumor cell enrichment. Most of the tumor cells in the samples were positive for cytokeratin and negative for vimentin, while the stromal cells showed the opposite pattern (Fig. S1). These suspensions were then separated into two fractions using only anti-keratin antibody-labeled microbeads. The populations considered to be tumor cells (cytokeratin+, vimentin−) were enriched in the tumor fractions compared to the unseparated samples, whereas in the residual fraction, these populations decreased in both diffuse-type and intestinal gastric cancers (Fig. 1A and Fig. S2). The residual and tumor fractions contained 0.80–12% (mean 4.4%) tumor cells and 0.37–4.1% (mean 1.6%) non-tumor cells (cytokeratin and vimentin+) as contaminants, respectively. Tumor cells increased by at least 1.5-fold in the tumor fraction compared to the unseparated samples for both diffuse-type and intestinal gastric cancer, whereas the percentage of tumor cells decreased in the residual fraction, resulting in significantly greater tumor enrichment in the tumor fraction than in the residual fraction (Fig. 1B). These results indicate that cytokeratin-expressing tumor cells from FFPE tissue sections of diffuse-type and intestinal gastric cancers were efficiently concentrated in the tumor fraction.

To evaluate whether NGS was performed correctly, we confirmed the quality of the DNA and NGS metrics. All fractionated samples had a DNA integrity number (DIN) greater than 2, average read depths greater than 1000, and no more than 60% duplication rate (Fig. S3). Therefore, NGS was considered to be performed correctly on the cell-isolated samples derived from FFPE tissue sections.

### Impact of tumor cell enrichment on CNV detection

**Figure 2:**
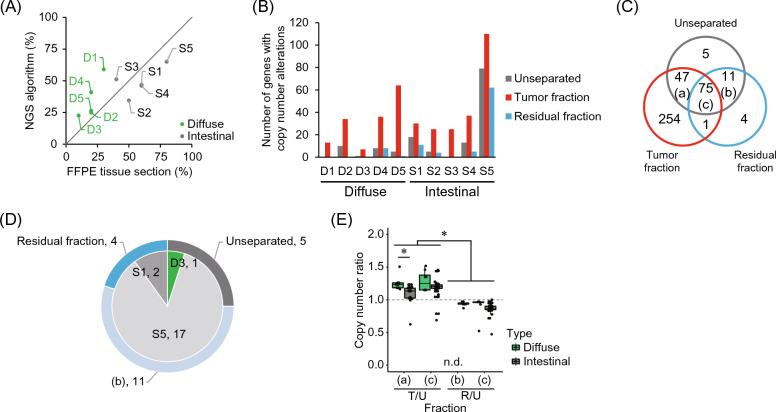
Influence of tumor cell enrichment on the detection of copy number variation (CNV). (**A**) Comparison of tumor content between evaluation using hematoxylin and eosin (H&E) staining and estimation using next generation sequencing (NGS). All evaluations of tumor content by HE staining were performed by a single pathologist. (**B**) Number of genes with CNV in separated fractions and unseparated sample. (**C**) Venn diagram of CNV detected in each fraction. (**D**) Samples with undetected CNVs in tumor fraction. (b) correspond to the common factor in the Venn diagram in (**C**). The breakdown of a total of 20 CNVs detected outside the tumor fraction (red circle) in (**C**) is represented by the outer circle. The sample composition in which these CNVs were found is indicated by the inner circle. (**E**) Copy number ratios commonly found in each fraction. (a)–(c) correspond to the common factor in the Venn diagram in (**C**). The copy number ratio is defined as the copy number in the tumor/residual fraction divided by that in the unseparated sample. *T/U* tumor/unseparated, *R/U* residual/unseparated, *n.d.* not detected. Asterisk represents the significance at *p* < 0.01.

In our study, no case of intestinal gastric cancer had a tumor content of less than 30% based on the pathologist’s histological evaluation, whereas diffuse-type gastric cancer had a tumor content of less than 30% (Fig. S1). Although a positive correlation was observed between this evaluation and NGS (*r* = 0.70, *p* < 0.01), no significant difference in tumor content was observed between cancer types (Fig. 2A). To investigate whether the enrichment of tumor cells using tissue suspension affected the detection of CNV, we identified genes with CNV using targeted sequencing of a panel of genes (225 genes listed in Table S1). The number of CNVs detected in the tumor fraction were higher than that in the unseparated and residual fractions (Fig. 2B). CNVs, including the loss of one allele, increased in the tumor fraction, suggesting that tumor enrichment specifically improved copy number loss detection (Fig. S4). Furthermore, 64% (254/397) of the CNVs were specific to the tumor fraction, and 5% (20/397) were detected only in the unseparated and residual fractions (Fig. 2C). Among the genes not detected in the tumor fraction, 85% (17/20) were derived from intestinal gastric cancer (S5), which had the highest estimated tumor content in this experiment (Fig. 2D). For the common CNVs obtained from the Venn diagram (Fig. 2C), the CNVs detected in the residual fraction were smaller than those in the unseparated sample. Thus, all CNV ratios were less than 1, whereas the ratios of common CNVs in the tumor fraction and unseparated samples were majorly greater than 1 (Fig. 2E). This suggest the residual fraction could be used as a normal reference.

### CNV evaluation using residual fractions as reference

**Figure 3:**
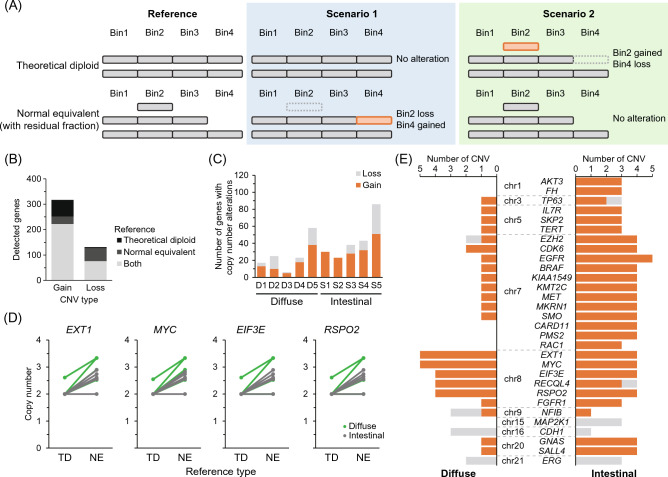
Copy number variation (CNV) analysis using residual fraction as reference. (**A**) Conceptual diagram of CNV evaluation by theoretical diploid reference without the blood sample and normal equivalent reference with the residual fraction. Two scenarios with different CNV results for the theoretical diploid and the normal equivalent reference are shown. In Scenario 1, CNV is not detected from each Bin in the theoretical diploid reference, while in the normal equivalent reference, loss of Bin2 and gain of Bin4 are detected. Scenario 2 shows an example where CNVs are not detected in the normal equivalent reference. (**B**) Number of genes with gain and loss by CNV analysis using the theoretical diploid and the normal equivalent references. (**C**) CNV analysis using the normal equivalent reference in each diffuse and intestinal gastric cancer. (**D**) Variations of CNV in four genes between the theoretical diploid (TD) and normal equivalent (NE) reference. Four genes with significantly altered CNV by using normal equivalent reference were extracted. (**E**) Genes with high frequency of CNVs detected in diffuse and intestinal type gastric adenocarcinoma. Genes with a gain or loss of less than 40% (2/5) in CNV analysis using normal equivalent reference were excluded. Chromosome numbers encoding genes are represented to the left of the gene symbol.

For CNV analyses without germline/normal references, such as those using blood (which contains normal cells), a homogeneous bin was assumed and set as a theoretical diploid reference based on the sequencing information of the target sample. A homogeneous bin assuming a neutral copy number (i.e. log2 of 0.0) was created using the “reference” tool in CNVkit, as guided in the manual (https://cnvkit.readthedocs.io/en/stable/pipeline.html). This was set as a theoretical diploid reference based on the sequencing information of the target sample. Therefore, an unexpected bias in read mapping may result in the detection of false alterations as a CNV even in its absence. To improve the accuracy of CNV detection, we attempted to detect CNV using a residual fraction enriched with normal cells instead of a germline/normal reference. A conceptual diagram of the CNV evaluation using a normal equivalent reference with the residual fraction is shown in Fig. 3A and compared with a theoretical diploid reference. Examples of CNVs not detected by the theoretical diploid reference (Scenario 1) and false alterations (Scenario 2) are presented. Comparing the CNV using the normal equivalent and theoretical diploid references, 31 gains and 51 losses were observed in the normal equivalent reference, whereas 65 gains and 4 losses, which were considered false alterations, were detected in the theoretical diploid reference (Fig. 3B). No differences were observed in the number of CNVs detected in each sample (Fig. 3C). The CNVs detected using this method are listed in Table S2. Four genes with significantly altered copy numbers were detected in the CNV analysis using the normal equivalent reference compared with the theoretical diploid reference, all of which were located on chromosome 8 (Fig. 3D). Furthermore, based on the Log R ratio, *MYC* copy number gains were observed in most exonic regions, except in one case (S5) of intestinal gastric cancer (Fig. S5). These results suggested that our targeted sequencing harbored an unexpected bias in the read mapping of chromosome 8; thus, the gain of chromosome 8 was not detected in the CNV analysis using the theoretical diploid reference. To compare CNVs between diffuse-type and intestinal gastric cancers, genes with alterations observed in more than 60% (3/5) of either cancer type were extracted (Fig. 3E). Using the normal equivalent reference assessed from the residual fraction, chromosomes 7 and 20 were gained less frequently in the diffuse-type than in the intestinal gastric cancer, while gain of chromosome 8 was observed in both types of cancer.

### Detection of gene amplification by tumor cell enrichment

**Figure 4:**
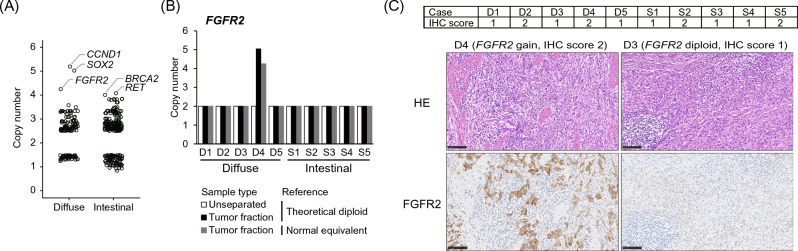
Copy number variation (CNV) and protein expression. (**A**) Copy number alterations extracted using normal equivalent references. Gene symbols with copy number > 4 in diffuse gastric cancer are indicated. (**B**) Comparison of CNV in FGFR2 between theoretical diploid and normal equivalent references. (**C**) Immunohistochemical analysis of FGFR2 protein expression. The expression level of each case was classified as low (score 1) and high (score 2) in the upper table. The representative photomicrographs of the FGFR2 expression in the lower figures. *HE* hematoxylin and eosin staining. Scale bars, 100 μm.

CNV analysis in diffuse-type gastric cancers, using the residual fraction as a normal equivalent reference, identified three genes (*CCND1*, *SOX2*, and *FGFR2*) with copy numbers > 4 (Fig. 4A). Amplification of *FGFR2*, which is attractive for targeted therapy via FGFR inhibition in gastric cancer^14^, was detected only in the tumor enrichment of case D4 (diffuse gastric cancer) (Fig. 4B). To examine FGFR2 protein expression, immunohistochemistry (IHC) was performed using a specific antibody against FGFR2. Overexpression (score 2) of FGFR2 protein was observed in four cases, including D4 (Fig. 4C and Fig. S6). Although FGFR2 staining showed mild staining (score 1) in normal gastric epithelium, four tumors (D2, D4, S2, and S5) showed extensive and strong staining (score 2, Fig. S7).

## Discussion

In the present study, cell enrichment was performed using anti-keratin antibodies, as previously reported, to detect somatic mutations^13^, resulting in the detection of an increased number of CNVs. Single-cell analysis of gastroesophageal tumors has revealed heterogeneous expression of the keratin gene family and EMT-related genes^15,16^. Notably, this enrichment method resulted in a significant increase in the number of CNVs detected, despite the fact that the use of anti-keratin antibodies can lead to a reduction in tumor enrichment due to tumor heterogeneity. Future identification of proteins that are less susceptible to tumor heterogeneity and enrichment of tumor cells with antibodies specific for these proteins may help further increase the number of CNVs detected.

Using this tumor cell enrichment method, the number of detected CNVs significantly increased; however, 5% of the alterations were no longer detectable. Most of these alterations were derived from a single sample with high tumor content. Assuming that there are fewer copy number alterations in normal tissue than in tumor cells, it is possible that in this case, owing to the heterogeneity of gastric cancer^15^, the relative tumor content in the residual fraction was increased by a certain number of cytokeratin-negative tumor cells that could not be enriched with the anti-cytokeratin antibody and were thus detected as CNVs in the residual fraction. Therefore, this enrichment method is considered unsuitable for samples with high tumor content. The copy number ratio (tumor fraction/unseparated) tended to be higher in diffuse-type gastric cancer than in intestinal gastric cancer, suggesting that samples with lower tumor content in histological examination were more affected by the enrichment and showed an improved sensitivity to CNV. Based on the above results, we conclude that our enrichment method has a positive impact on CNV detection, especially in FFPE tissue sections with low tumor content.

In the CNV evaluation using a theoretical diploid reference as a control, only a gain was detected in the residual fraction, where most tumor cells were removed (Fig. S4). This implies that CNV analysis using hypothetical controls overestimates copy number gain. In support of this, a greater gain was detected in the theoretical diploid reference (Fig. 3B). In panel sequencing, it is difficult to prevent contamination by false gains using CNV analysis with theoretical diploid references. In contrast, CNV analysis using normal equivalent references detected both copy number gains and losses. Because the normal equivalent reference consists of a residual fraction dominated by non-cancerous cells, it is expected to strongly reflect the read mapping of the germline/normal reference. Therefore, these alterations were considered underestimated CNVs.

Recently, whole genome analysis of diffuse-type and intestinal gastric cancers has been performed^17^. Amplification of chromosome 8 in diffuse-type gastric cancer and chromosomes 7, 8, and 20 in intestinal gastric cancer was observed at high frequencies. These results are consistent with the CNV results obtained in this study, using the normal equivalent reference. Additionally, the loss of CDH1, observed in whole-genome analysis of enriched cells from the ascitic fluid of peritoneal metastasis^18^, was also detected in the CNV analysis of diffuse gastric cancer. Our analysis yielded CNVs consistent with those of the whole-genome analysis in previous report^17^, even in FFPE tissue sections with low tumor content. CNV evaluation using a normal equivalent reference can extract alterations in the bin that would be missed by a theoretical diploid reference, resulting in improved loss detection sensitivity and reduced false gains.

Tumor tissues with copy number gain in the target gene are occasionally negative for the detection of proteins translated from that gene by IHC. The concordance rate between equivocal amplification of a chromosome and positive immunostaining is important for evaluating the performance of panel sequencing. Therefore, we performed immunostaining for FGFR2, in which CNVs were detected by tumor enrichment. The majority of tumor cells with *FGFR2* amplification were stained, implying that the protein was overexpressed due to this chromosomal alteration. On the other hand, since FGFR2 protein expression without chromosomal amplification can also be caused by aberrant gene expression or a fusion gene^19^, we speculated that the three IHC-positive tumors with *FGFR2* diploid expressed FGFR2 protein due to factors other than amplification. Although further validation is needed, our results suggest that CNV analysis using the residual fraction as a normal equivalent reference may improve the concordance between chromosomal amplification and protein expression in diffuse-type gastric cancer.

In this study, NGS was performed in 10 gastric cancers to demonstrate an improvement in the number of CNVs using tumor cell enrichment. Further evaluation including IHC with a larger sample size would be needed for validation of CNV detection. In addition, a comparison of tumor enrichment with typical enrichment methods using macrodissectable tumors will be performed in the future to demonstrate the superiority of this tumor enrichment.

In the present study, we developed a new approach for CNV analysis using tumor cell enrichment to improve the detection of CNV in target panel sequencing of FFPE tissue sections with low tumor content. The enrichment of sporadic tumor cells in diffuse-type gastric cancer with low tumor content increases the number of CNVs detected via NGS and contributes to an improvement in copy number. On the other hand, this enrichment method was considered unsuitable for CNV detection in cases with high tumor content. Furthermore, CNV analysis using the residual fraction as a convenient reference instead of a germline/normal reference allowed panel sequencing to detect the CNV features of diffuse-type gastric cancer. Our approach will not only contribute to increasing the detection rate of CNV in target panel sequencing through tumor enrichment, but also has promising prospects for improving the accuracy of CNV detection in archived specimens without paired blood samples.

## Methods

### Ethical statement

All aspects of this study were approved by the Institutional Review Board of Shizuoka Cancer Center (authorization number 25-33). In this study, pathogenic germline alterations were unintentionally predicted in retrospective FFPE specimens. To avoid disadvantaging specimen donors, appropriate informed consent with the approval of the Ethics Review board, including the possibility of secondary findings, such as those found in blood-based constitutional analyses, was obtained. Human studies followed the ethical guidelines for clinical application, in accordance with the Declaration of Helsinki.

### Clinical samples

FFPE samples from 10 patients with gastric cancer were obtained from the tissue bank of the Division of Pathology at the Shizuoka Cancer Center (Table S3). Tissue sections from five diffuse and five intestinal gastric cancer cases were collected between 2014 and 2019.

### Suspension and isolation of FFPE tissue samples

A FFPE tissue block of gastric cancers was cut into 10 μm thick sections. These sections were dewaxed for 10 min by incubating thrice in xylene followed by sequential rehydration by 30 s of incubation in each of the following dilutions of ethanol: 100% (twice), 70%, 50%, and 30%. The hydration process was completed with 30 s of incubation in deionized water. The dewaxed samples were suspended using a gentleMACS Octo Dissociator with Heaters (program, 37C_FFPE_1; Miltenyi Biotec, Bergisch Gladbach, Germany), and heat-induced antigen retrieval was performed according to the manufacturer’s protocol.

Fully automated cell labeling and separation were performed using an autoMACS Pro Separator (Miltenyi Biotec) according to the manufacturer’s protocol. The cell suspensions derived from FFPE tissue sections were separated using Anti-Cytokeratin MicroBeads (Miltenyi Biotec) and stained using anti-cytokeratin-FITC (clone REA831, Miltenyi Biotec), anti-vimentin-APC (clone REA409, Miltenyi Biotec), and CD235a (Glycophorin A)-PE (clone REA175, Miltenyi Biotec) antibodies. The nuclei were stained with the DAPI Staining Solution (Miltenyi Biotec).

### Targeted sequencing of the gene panel

DNA was extracted from FFPE tissue and peripheral blood samples using the GeneRead DNA FFPE Kit and QIAamp DNA blood Mini Kit (Qiagen, Venlo, Nederland), respectively. Purified DNA was quantified using a NanoDrop and a Qubit 2.0 Fluorometer (Thermo Fisher Scientific, Waltham, MA, USA). To check the quality of the DNA, DIN was determined using a TapeStation (Agilent Technologies, Santa Clara, CA, USA). A SureSelect Custom panel kit (Agilent) was used to enrich the 225 cancer-related genes listed in Table S1 by hybridization-based target capturing. Target capture and sequencing were performed using a clinical testing platform, “the 225S Cancer Panel”, at the SRL&S Higoka Cancer Center Collaborative Laboratories, Inc. (Shizuoka, Japan), a CAP-accredited and CLIA-certified laboratory. In total, 2.427 Mb of the human genome, including 0.723 Mb of exon regions of the RefSeq genes, were captured, sequenced, and delivered in the standard FASTQ format. The reads were aligned to the reference human genome (UCSC hg19) and subjected to further analyses. In total, 2.427 Mb of the human genome, including 0.723 Mb exon regions of RefSeq genes, were encompassed by 55,765 biotinylated RNA oligomers (120 bp in length). Raw binary data derived from the sequencer were converted into sequence reads using bcl2fastq (ver. 2.20, Illumina), and mapped to the reference human genome (UCSC hg19).

### Evaluation of CNV using NGS

CNVs were detected using CNVkit (version 0.9.9)^20^. In the paired analysis, the “autobin” tool was first applied to define target bins with the options “-m hybrid --target-min-size {60, 120, 240} -g access-5k-mappable. hg19.bed”. The “coverage” tool was used with the default options to calculate target coverage in both tumor and normal-equivalent (residue after tumor enrichment) samples. The “reference” tool was then applied to each pair with the target and anti-target regions calculated in the previous steps. The " “fix”, and “segment” tools were applied sequentially to each pair with the “-y” and “--drop-low-coverage -m cbs” options added to the “fix” and “segment” tools, respectively. For non-paired analysis, the “autobin” tool was used as described above, followed by the “batch” tool with the “--method hybrid --y --drop-low-coverage --segment-method cbs” options. The “call” tool was then run with the “-m clonal -y” options. The resulting output was exported in the standard BED and VCF formats using the “export” tool with the options “--show all -y” and “--y -i {the name of a tumor sample}” for BED and VCF, respectively. In both modes, the resulting CNV kit outputs (raw results) were combined and stored in the standard VCF format using a combination of standard Linux command line tools (tail, awk, sort, uniq, sed, and join). Results were annotated using AnnotSV (version 2.4)^21^.

### Immunohistochemical analysis

Specimens used for the CNV analysis were also used for immunohistochemical staining. Immunostaining was performed with an automated stainer (Leica Bond-III, Leica, Tokyo, Japan) using a heat-based antigen retrieval technique with BOND Epitope Retrieval Solution 1 for 20 min at 100 °C according to the manufacturer's protocol. A mouse monoclonal antibody against human FGFR2 (ab58201; Abcam, Cambridge, UK) was used at a dilution of 500. FGFR2 expression was scored using the intensity of staining and the percentage of stained tumor cells as follows: Score 1, < 80% of tumor cells strongly expressed, and Score 2, ≥ 80% of tumor cells strongly expressed. Strong FGFR2 expression was scored when cytoplasmic or membranous staining was stronger than that in normal gastric epithelium in the same section.

### Statistical analysis

Significant differences were determined using the Welch’s *t*-test. *P*-value < 0.01 was considered significant.

### Supplementary Information

Supplementary Information.

## Data Availability

The authors declare that all other data supporting the findings of this study are available within the article and its supplementary information files, and from the corresponding author upon reasonable request.
