# Supplementary material for: Tumor cell enrichment by tissue suspension improves sensitivity to copy number variation in diffuse gastric cancer with low tumor content
Source: Sci Rep. 2024 Jun 13;14:13699. doi: 10.1038/s41598-024-64541-3 (PMC11176319; doi:10.1038/s41598-024-64541-3)
Supplement: Supplementary file 1 — Supplementary Information. [file 41598_2024_64541_MOESM1_ESM.docx]

**Tumor cell enrichment by tissue suspension improves sensitivity to copy number variation in diffuse gastric cancer with low tumor content**

Keiichi Hatakeyama^1,*^, Koji Muramatsu^2^, Takeshi Nagashima^3,4^, Hiroyuki Ichida^5^, Yuichi Kawanishi^5^, Ryutaro Fukumura^5^, Keiichi Ohshima^6^, Yuji Shimoda^3^, Sumiko Ohnami^3^, Shumpei Ohnami^3^, Koji Maruyama^7^, Akane Naruoka^8^, Hirotsugu Kenmotsu^9^, Kenichi Urakami^3^, Yasuto Akiyama^10^, Takashi Sugino^2^ and Ken Yamaguchi^11^

^1^Cancer Multiomics Division, Shizuoka Cancer Center Research Institute, Sunto-gun, Shizuoka 411-8777, Japan

^2^Division of Pathology, Shizuoka Cancer Center, Sunto-gun, Shizuoka 411-8777, Japan

^3^Cancer Diagnostics Research Division, Shizuoka Cancer Center Research Institute, Sunto-gun, Shizuoka 411-8777, Japan

^4^SRL Inc., Shinjuku-ku, Tokyo 163-0409, Japan

^5^SRL & Shizuoka Cancer Center Collaborative Laboratories Inc., Sunto-gun, Shizuoka 411-8777, Japan

^6^Medical Genetics Division, Shizuoka Cancer Center Research Institute, Sunto-gun, Shizuoka 411-8777, Japan

^7^Experimental Animal Facility, Shizuoka Cancer Center Research Institute, Sunto-gun, Shizuoka 411-8777 Japan.

^8^Drug Discovery and Development Division, Shizuoka Cancer Center Research Institute, Sunto-gun, Shizuoka 411-8777 Japan.

^9^Division of Thoracic Oncology, Shizuoka Cancer Center, Sunto-gun, Shizuoka 411-8777, Japan

^10^Immunotheraphy Division, Shizuoka Cancer Center Research Institute, Sunto-gun, Shizuoka 411-8777, Japan

^11^Shizuoka Cancer Center, Sunto-gun, Shizuoka 411-8777, Japan

* Correspondence should be addressed to Keiichi Hatakeyama (email: k.hatakeyama@scchr.jp; phone: +81-55-989-5222; fax: +81-55-989-6085)

**
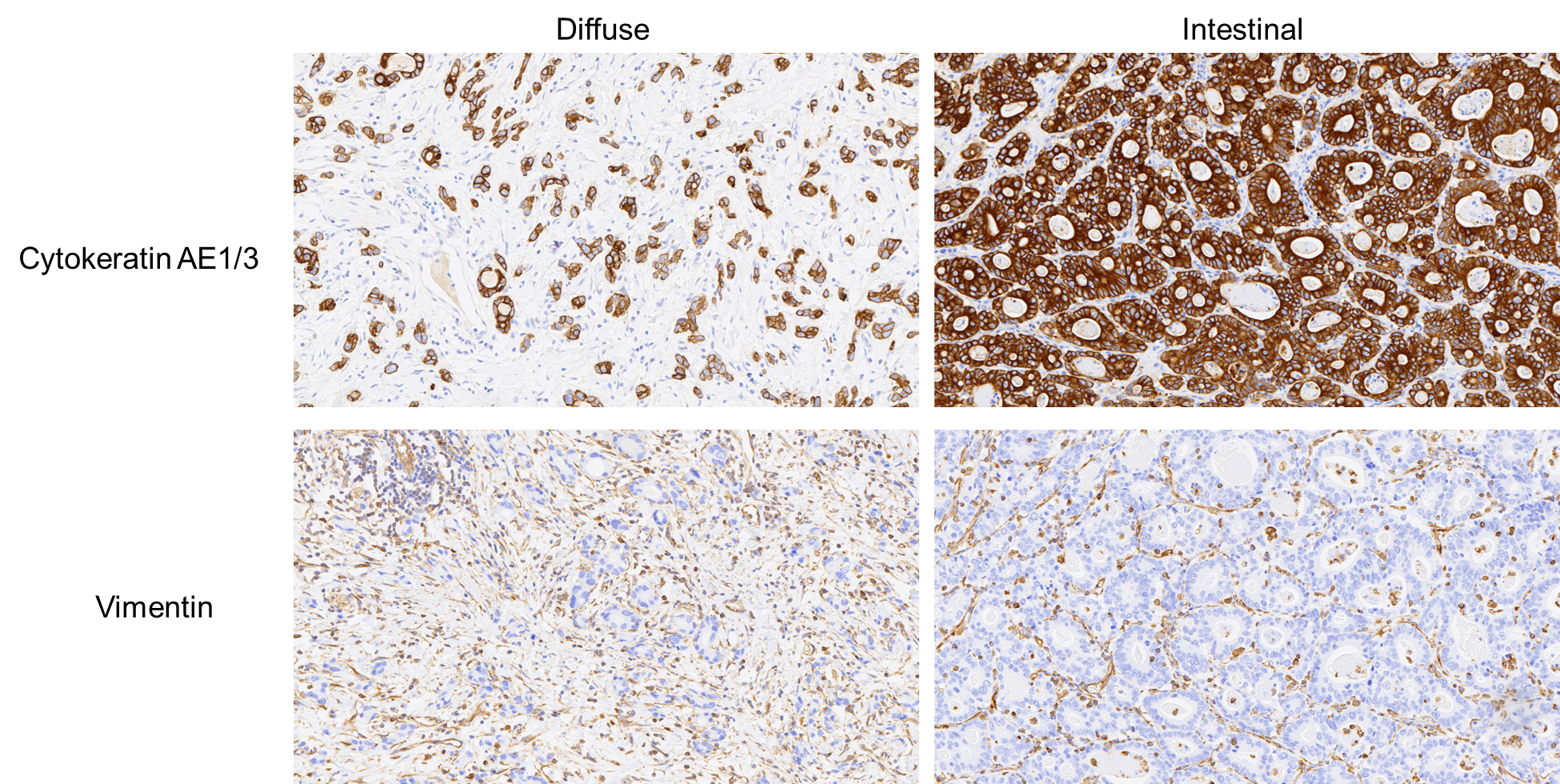

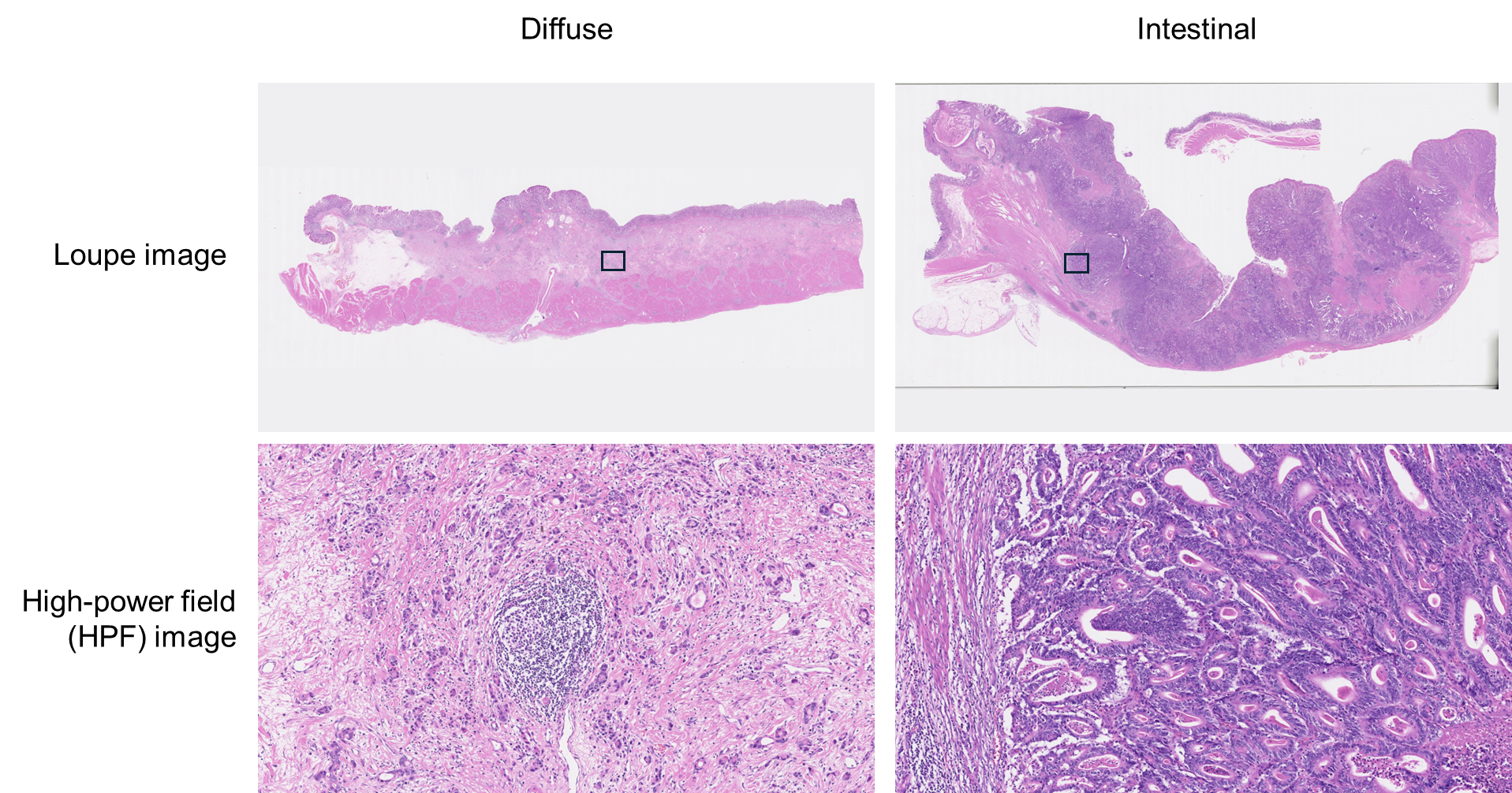
**

**Supplementary Figure S1.** **Representative hematoxylin and eosin (HE) staining and immunehistochemical (IHC) images of cytokeratin and vimentin in the diffuse-type and intestinal gastric cancers.** The top four images are HE stained images and the bottom four images are IHC images. The anti-cytokeratin antibody cocktail (AE1/3) can stain both acidic cytokeratins (type I) and basic cytokeratins (type II).

**
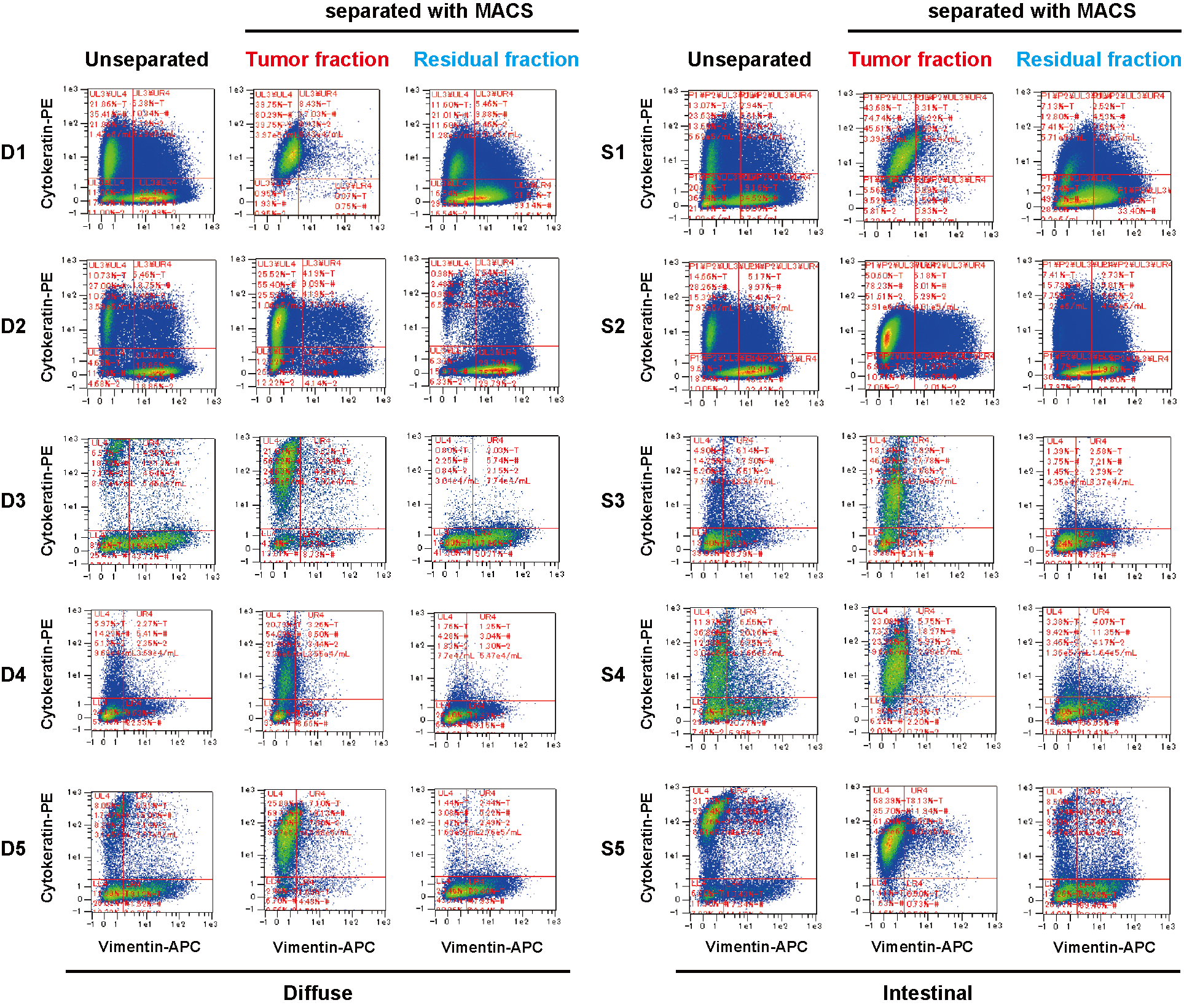
**

**Supplementary Figure S2.** **Flow cytometry of formalin-fixed paraffin-embedded (FFPE) tissue sections.** The FFPE tissue sections were suspended and separated using magnetic-activated cell sorting (MACS) with anti-cytokeratin microbeads. Thereafter, these fractions were stained with anti-cytokeratin and vimentin antibodies. To distinguish nuclei and erythrocytes, DAPI and CD235 staining were simultaneously performed. The suspensions enriched with the microbeads are defined as tumor fractions, and samples that could not be captured with these beads are designated as residual fractions. The suspensions that were not subjected to MACS are represented as unseparated.

**
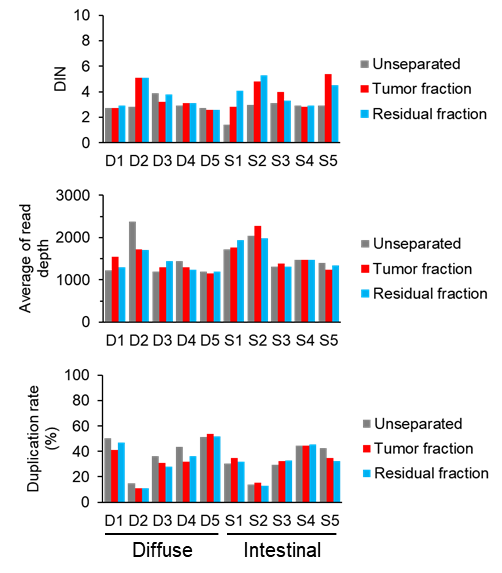
**

**Supplementary Figure S3.** **Quality of extracted DNA and sequencing metrics.** DNA integrity number (DIN) (top panel), read depth (middle panel), and duplication rate (bottom panel) in diffuse and intestinal gastric cancer. Tissue suspensions enriched with microbeads are defined as tumor fractions, and samples that could not be captured with these beads are designated as residual fractions. FFPE tissue sections that were not subjected to tumor cell enrichment are represented as unseparated.

**
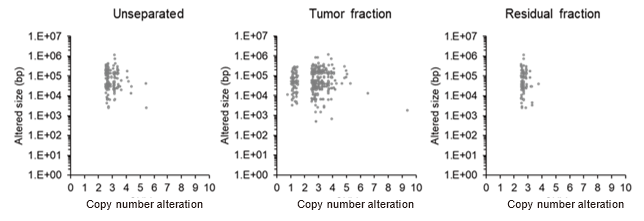
**

**Supplementary Figure S4.** **Distribution of copy number alteration and altered region sizes in each fraction.** Target regions with no copy number alterations (non-CNV range, 1.5–2.5) were excluded from the plot.


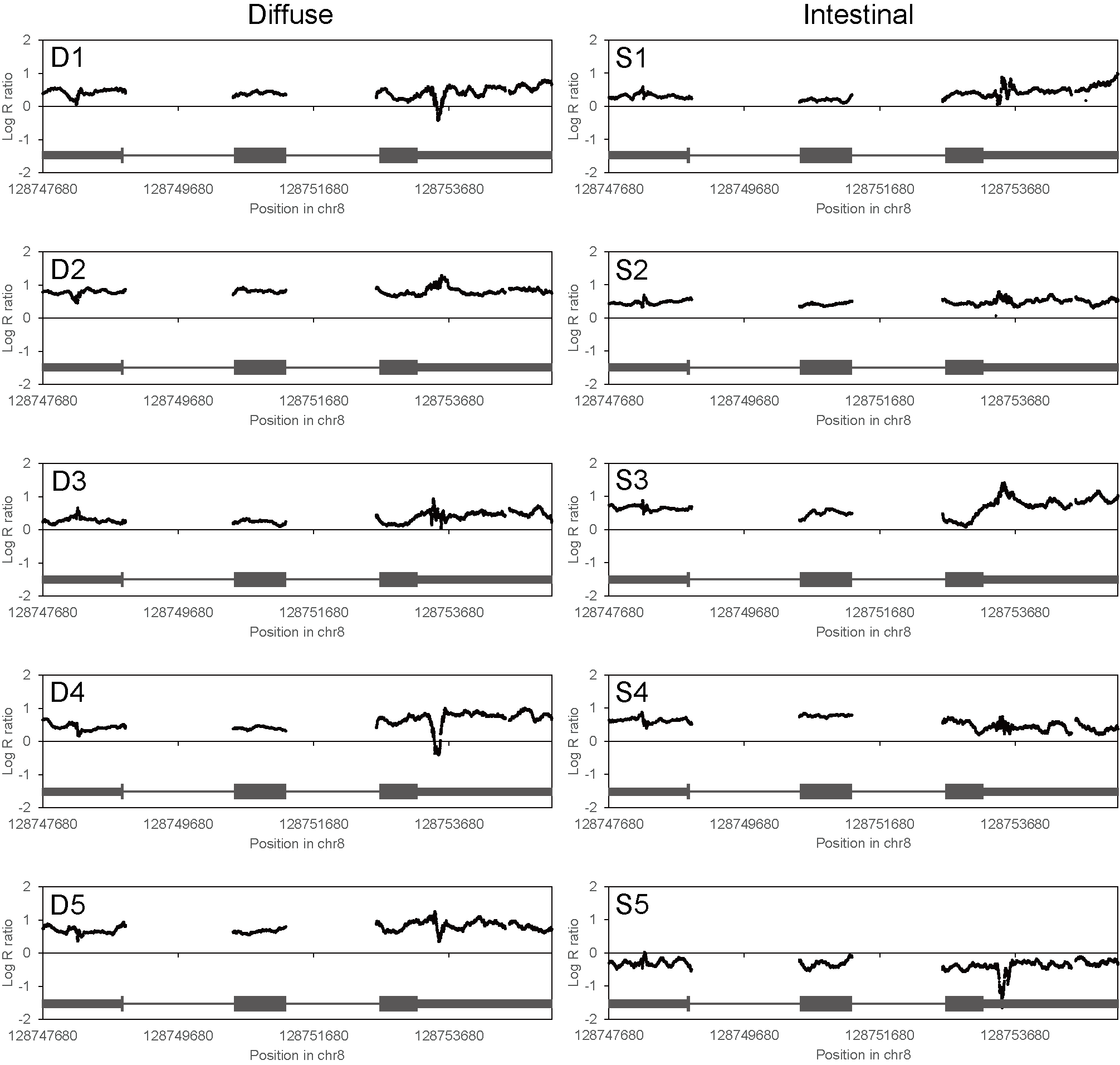


**Supplementary Figure S5.** **Log R ratio of *MYC* gene using normal equivalent reference.** The Log R ratio was defined as the normalized read depth of the tumor fraction divided by the normal equivalent reference as control. Exonic regions encoding the *MYC* gene are indicated by gray boxes.

**
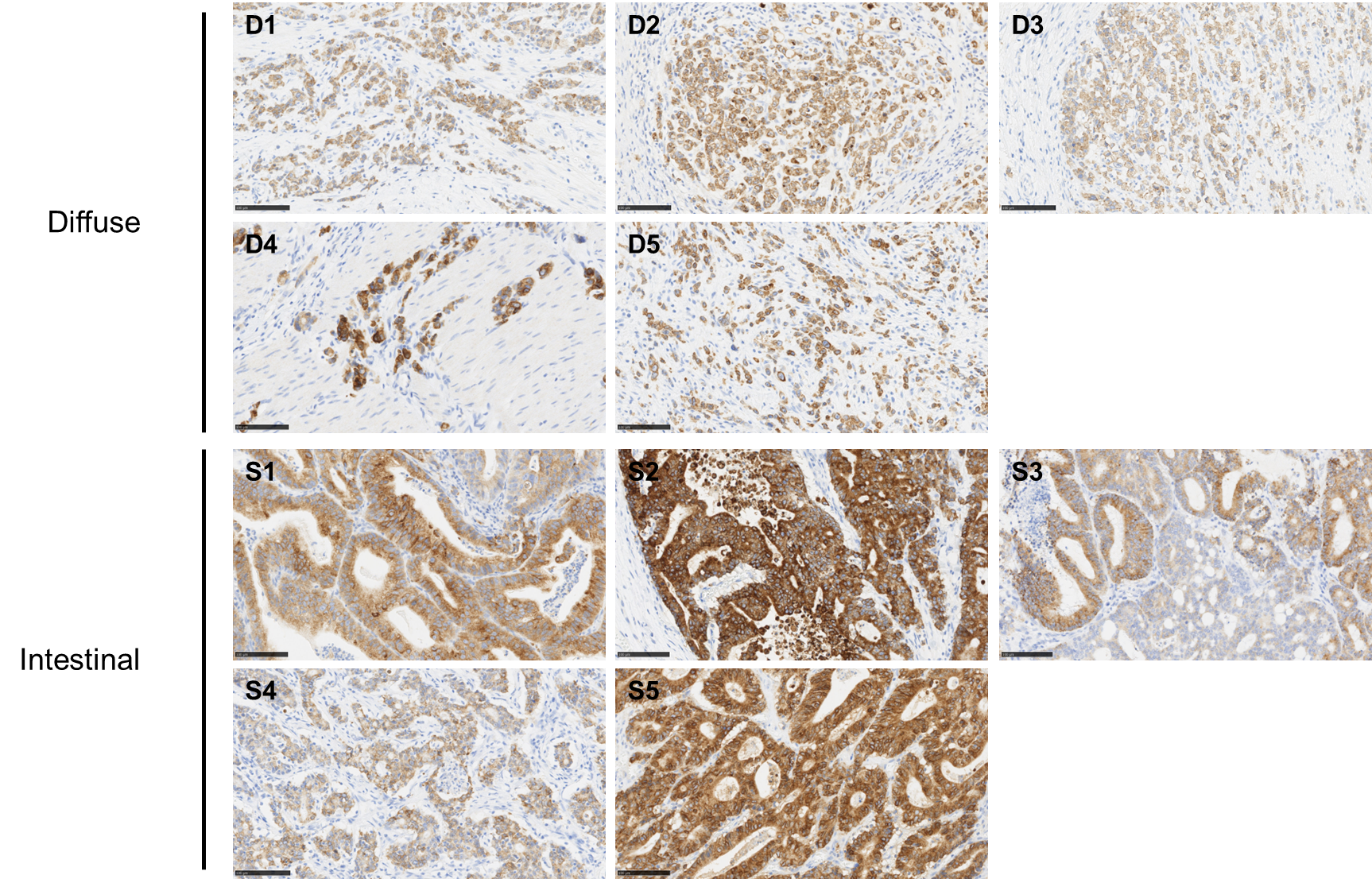
**

**Supplementary Figure S6.** **Immunehistochemical images of FGFR2 in the diffuse-type and intestinal gastric cancers.**

**
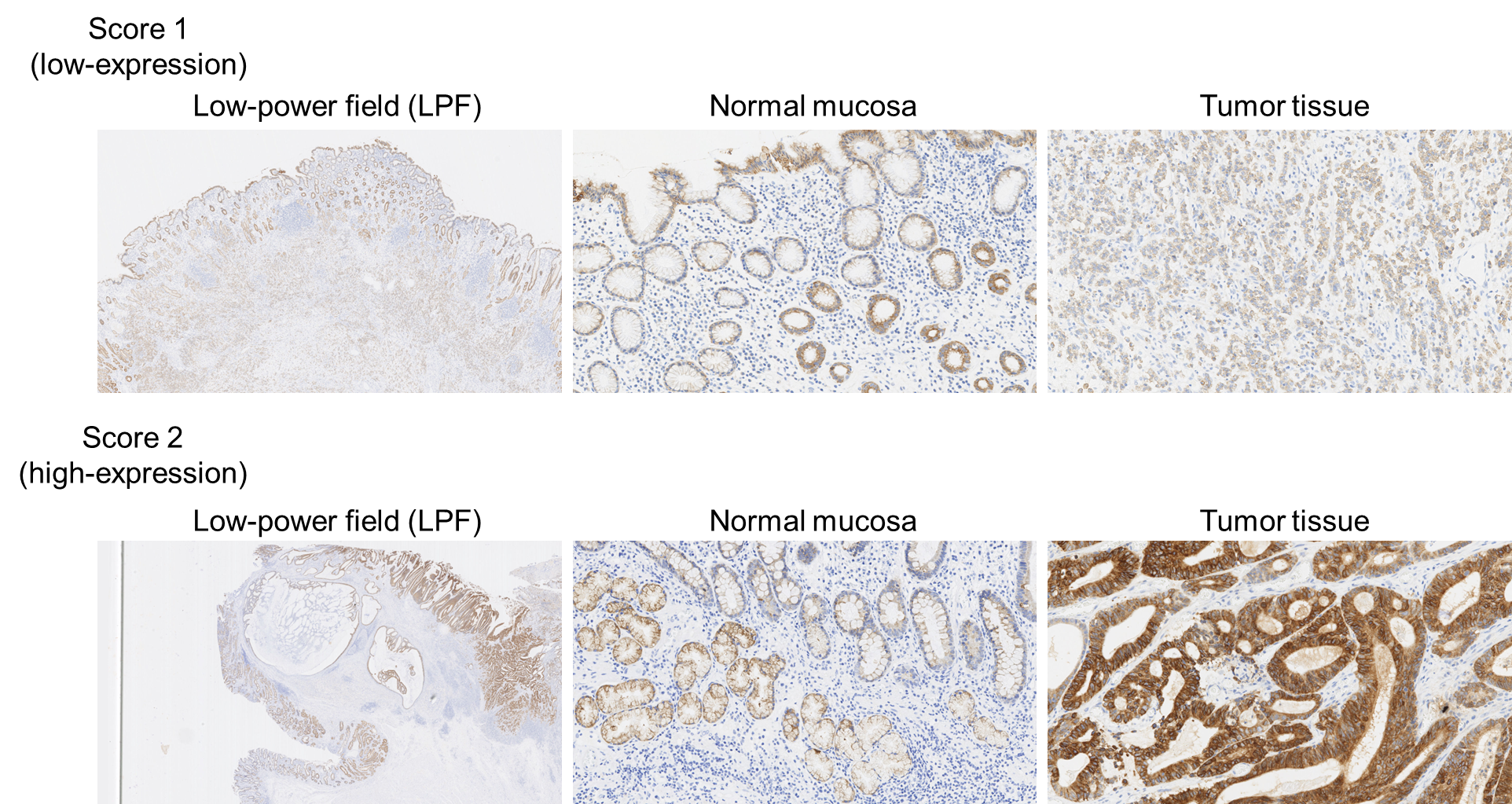
**

**Supplementary Figure S7.** **Representative immunohistochemical (IHC) images of FGFR2 in normal epithelium derived from sections of diffuse-type and intestinal gastric cancers.**

**Supplementary Table S1. List of 225 genes in target panel sequencing**

| Target gene (225 genes) | | | | | | | |
| --- | --- | --- | --- | --- | --- | --- | --- |
| *ABL1* | *CCND1* | *ENG* | *IDH1* | *MITF* | *PDGFRA* | *SDHAF2* | *TSC1* |
| *ACTN4* | *CD274* | *ENO1* | *IGF1R* | *MKRN1* | *PDGFRB* | *SDHB* | *TSC2* |
| *ACVR1B* | *CD74* | *EP300* | *IGF2* | *MLH1* | *PHOX2B* | *SDHC* | *TSHR* |
| *AKT1* | *CDC73* | *EPAS1* | *IL7R* | *MSH2* | *PIK3CA* | *SDHD* | *U2AF1* |
| *AKT2* | *CDH1* | *ERBB2* | *IRF4* | *MSH6* | *PIK3R1* | *SETD2* | *UGT1A1* |
| *AKT3* | *CDK4* | *ERBB3* | *JAK1* | *MTOR* | *PIK3R2* | *SF3B1* | *VHL* |
| *ALK* | *CDK6* | *ERBB4* | *JAK2* | *MUTYH* | *PMS2* | *SH2D1A* | *VTI1A* |
| *AMER1* | *CDKN1A* | *ERG* | *JAK3* | *MYB* | *POLD1* | *SKP2* | *WT1* |
| *APC* | *CDKN1B* | *ESR1* | *JUN* | *MYC* | *POLE* | *SMAD2* |  |
| *AR* | *CDKN2A* | *EXT1* | *KDM5C* | *MYCL* | *PPP2R1A* | *SMAD4* |  |
| *ARAF* | *CDKN2B* | *EXT2* | *KDM6A* | *MYCN* | *PRDM1* | *SMARCA4* |  |
| *ARID1A* | *CDKN2C* | *EZH2* | *KEAP1* | *MYD88* | *PRKAR1A* | *SMARCB1* |  |
| *ARID1B* | *CHEK2* | *EZR* | *KIAA1549* | *NCOA3* | *PRKCI* | *SMO* |  |
| *ARID2* | *CIC* | *FANCC* | *KIF1B* | *NCOA4* | *PTCH1* | *SOX2* |  |
| *ATM* | *COL1A1* | *FAT1* | *KIF5B* | *NCOR1* | *PTEN* | *SOX9* |  |
| *ATRX* | *CREBBP* | *FBXW7* | *KIT* | *NF1* | *PTPRK* | *SPOP* |  |
| *AXIN1* | *CRKL* | *FGFR1* | *KLF4* | *NF2* | *RAC1* | *STAG2* |  |
| *AXL* | *CRLF2* | *FGFR2* | *KMT2C* | *NFE2L2* | *RAC2* | *STAT3* |  |
| *B2M* | *CSF1R* | *FGFR3* | *KRAS* | *NFIB* | *RAD51C* | *STK11* |  |
| *BAP1* | *CTCF* | *FGFR4* | *LMO1* | *NKX2-1* | *RAF1* | *STRN* |  |
| *BARD1* | *CTLA4* | *FH* | *MAP2K1* | *NOTCH1* | *RB1* | *TACC3* |  |
| *BAX* | *CTNNB1* | *FLCN* | *MAP2K4* | *NOTCH2* | *RECQL4* | *TCF7L2* |  |
| *BCL10* | *CUL3* | *FOXL2* | *MAP3K1* | *NOTCH3* | *RET* | *TEK* |  |
| *BCL2L11* | *CYLD* | *FUBP1* | *MAP3K4* | *NRAS* | *RHOA* | *TERT* |  |
| *BMPR1A* | *DAXX* | *G6PD* | *MAPK1* | *NRG1* | *RNF43* | *TMEM127* |  |
| *BRAF* | *DDR2* | *GATA3* | *MAX* | *NTRK1* | *ROS1* | *TMPRSS2* |  |
| *BRCA1* | *DNMT1* | *GNA11* | *MDM2* | *NTRK2* | *RRAS2* | *TP53* |  |
| *BRCA2* | *DPYD* | *GNAQ* | *MDM4* | *NTRK3* | *RSPO2* | *TP63* |  |
| *CARD11* | *EGFR* | *GNAS* | *MED12* | *PALB2* | *RSPO3* | *TPM3* |  |
| *CASP8* | *EIF3E* | *HNF1A* | *MEN1* | *PBRM1* | *SALL4* | *TPMT* |  |
| *CCDC6* | *EML4* | *HRAS* | *MET* | *PDGFB* | *SDC4* | *TRAF7* |  |

**Supplementary Table S2. List of copy number altered genes excluding copy number variation (CNV) overestimated candidates**

| Sample ID | Type | Gene symbol | Chromosome | RefSeq ID | CNV |
| --- | --- | --- | --- | --- | --- |
| D2 | Diffuse | *FGFR3* | chr4 | NM_000142 | 1.30 |
| D2 | Diffuse | *PHOX2B* | chr4 | NM_003924 | 1.30 |
| D2 | Diffuse | *TACC3* | chr4 | NM_006342 | 1.30 |
| D2 | Diffuse | *FAT1* | chr4 | NM_005245 | 1.48 |
| D2 | Diffuse | *FBXW7* | chr4 | NM_033632 | 1.48 |
| D2 | Diffuse | *EZR* | chr6 | NM_003379 | 1.31 |
| D2 | Diffuse | *MAP3K4* | chr6 | NM_005922 | 1.31 |
| D2 | Diffuse | *EZH2* | chr7 | NM_001203247 | 1.31 |
| D2 | Diffuse | *EIF3E* | chr8 | NM_001568 | 3.39 |
| D2 | Diffuse | *EXT1* | chr8 | NM_000127 | 3.39 |
| D2 | Diffuse | *MYC* | chr8 | NM_002467 | 3.24 |
| D2 | Diffuse | *RECQL4* | chr8 | NM_004260 | 3.39 |
| D2 | Diffuse | *RSPO2* | chr8 | NM_178565 | 3.39 |
| D2 | Diffuse | *NFIB* | chr9 | NM_005596 | 1.32 |
| D2 | Diffuse | *CCND1* | chr11 | NM_053056 | 5.54 |
| D2 | Diffuse | *ATM* | chr11 | NM_000051 | 1.40 |
| D2 | Diffuse | *SDHD* | chr11 | NM_003002 | 1.40 |
| D2 | Diffuse | *BRCA2* | chr13 | NM_000059 | 1.37 |
| D2 | Diffuse | *RB1* | chr13 | NM_000321 | 1.37 |
| D2 | Diffuse | *FLCN* | chr17 | NM_144997 | 1.27 |
| D2 | Diffuse | *NCOR1* | chr17 | NM_006311 | 1.27 |
| D2 | Diffuse | *GNAS* | chr20 | NM_080425 | 3.11 |
| D2 | Diffuse | *NCOA3* | chr20 | NM_181659 | 3.11 |
| D2 | Diffuse | *SALL4* | chr20 | NM_020436 | 3.11 |
| D2 | Diffuse | *SDC4* | chr20 | NM_002999 | 3.11 |
| D2 | Diffuse | *ERG* | chr21 | NM_001243428 | 1.25 |
| S1 | Intestinal | *FOXL2* | chr3 | NM_023067 | 2.89 |
| S1 | Intestinal | *PIK3CA* | chr3 | NM_006218 | 2.89 |
| S1 | Intestinal | *PRKCI* | chr3 | NM_002740 | 2.89 |
| S1 | Intestinal | *SOX2* | chr3 | NM_003106 | 2.89 |
| S1 | Intestinal | *TP63* | chr3 | NM_003722 | 2.89 |
| S1 | Intestinal | *CARD11* | chr7 | NM_001324281 | 2.67 |
| S1 | Intestinal | *EGFR* | chr7 | NM_005228 | 2.67 |
| S1 | Intestinal | *PMS2* | chr7 | NM_000535 | 2.67 |
| S1 | Intestinal | *RAC1* | chr7 | NM_006908 | 2.67 |
| S1 | Intestinal | *CDK6* | chr7 | NM_001145306 | 2.65 |
| S1 | Intestinal | *MET* | chr7 | NM_000245 | 2.90 |
| S1 | Intestinal | *KIAA1549* | chr7 | NM_001164665 | 2.60 |
| S1 | Intestinal | *MKRN1* | chr7 | NM_013446 | 2.60 |
| S1 | Intestinal | *SMO* | chr7 | NM_005631 | 2.60 |
| S1 | Intestinal | *EZH2* | chr7 | NM_001203247 | 2.68 |
| S1 | Intestinal | *KMT2C* | chr7 | NM_170606 | 2.68 |
| S1 | Intestinal | *FGFR1* | chr8 | NM_023110 | 2.61 |
| S1 | Intestinal | *EIF3E* | chr8 | NM_001568 | 2.61 |
| S1 | Intestinal | *EXT1* | chr8 | NM_000127 | 2.61 |
| S1 | Intestinal | *MYC* | chr8 | NM_002467 | 2.55 |
| S1 | Intestinal | *RECQL4* | chr8 | NM_004260 | 2.61 |
| S1 | Intestinal | *BRCA2* | chr13 | NM_000059 | 2.81 |
| S1 | Intestinal | *RB1* | chr13 | NM_000321 | 2.81 |
| S1 | Intestinal | *GNAS* | chr20 | NM_080425 | 2.55 |
| S1 | Intestinal | *NCOA3* | chr20 | NM_181659 | 2.55 |
| S1 | Intestinal | *SALL4* | chr20 | NM_020436 | 2.55 |
| S1 | Intestinal | *SDC4* | chr20 | NM_002999 | 2.55 |
| S2 | Intestinal | *CARD11* | chr7 | NM_001324281 | 2.79 |
| S2 | Intestinal | *EGFR* | chr7 | NM_005228 | 2.79 |
| S2 | Intestinal | *PMS2* | chr7 | NM_000535 | 2.79 |
| S2 | Intestinal | *RAC1* | chr7 | NM_006908 | 2.79 |
| S2 | Intestinal | *BRAF* | chr7 | NM_004333 | 2.76 |
| S2 | Intestinal | *CDK6* | chr7 | NM_001145306 | 2.76 |
| S2 | Intestinal | *EZH2* | chr7 | NM_001203247 | 2.76 |
| S2 | Intestinal | *KIAA1549* | chr7 | NM_001164665 | 2.76 |
| S2 | Intestinal | *KMT2C* | chr7 | NM_170606 | 2.76 |
| S2 | Intestinal | *MET* | chr7 | NM_000245 | 2.76 |
| S2 | Intestinal | *MKRN1* | chr7 | NM_013446 | 2.76 |
| S2 | Intestinal | *SMO* | chr7 | NM_005631 | 2.76 |
| S2 | Intestinal | *FGFR1* | chr8 | NM_023110 | 2.80 |
| S2 | Intestinal | *EIF3E* | chr8 | NM_001568 | 2.73 |
| S2 | Intestinal | *EXT1* | chr8 | NM_000127 | 2.73 |
| S2 | Intestinal | *MYC* | chr8 | NM_002467 | 2.66 |
| S2 | Intestinal | *RECQL4* | chr8 | NM_004260 | 2.73 |
| S2 | Intestinal | *GNAS* | chr20 | NM_080425 | 2.68 |
| S2 | Intestinal | *NCOA3* | chr20 | NM_181659 | 2.68 |
| S2 | Intestinal | *SALL4* | chr20 | NM_020436 | 2.68 |
| S2 | Intestinal | *SDC4* | chr20 | NM_002999 | 2.68 |
| D3 | Diffuse | *NRG1* | chr8 | NM_013964 | 2.64 |
| D3 | Diffuse | *EIF3E* | chr8 | NM_001568 | 2.55 |
| D3 | Diffuse | *EXT1* | chr8 | NM_000127 | 2.55 |
| D3 | Diffuse | *RSPO2* | chr8 | NM_178565 | 2.55 |
| D4 | Diffuse | *CDKN2C* | chr1 | NM_001262 | 2.63 |
| D4 | Diffuse | *JAK1* | chr1 | NM_001321853 | 2.63 |
| D4 | Diffuse | *JUN* | chr1 | NM_002228 | 2.63 |
| D4 | Diffuse | *MUTYH* | chr1 | NM_012222 | 2.63 |
| D4 | Diffuse | *MYCL* | chr1 | NM_001033081 | 2.62 |
| D4 | Diffuse | *BCL10* | chr1 | NM_003921 | 2.69 |
| D4 | Diffuse | *DPYD* | chr1 | NM_000110 | 2.69 |
| D4 | Diffuse | *NOTCH2* | chr1 | NM_024408 | 2.62 |
| D4 | Diffuse | *PIK3CA* | chr3 | NM_006218 | 3.27 |
| D4 | Diffuse | *PRKCI* | chr3 | NM_002740 | 3.27 |
| D4 | Diffuse | *SOX2* | chr3 | NM_003106 | 5.56 |
| D4 | Diffuse | *CDK6* | chr7 | NM_001145306 | 3.65 |
| D4 | Diffuse | *EXT1* | chr8 | NM_000127 | 2.91 |
| D4 | Diffuse | *MYC* | chr8 | NM_002467 | 2.82 |
| D4 | Diffuse | *RECQL4* | chr8 | NM_004260 | 2.91 |
| D4 | Diffuse | *FGFR2* | chr10 | NM_000141 | 4.28 |
| D4 | Diffuse | *ATM* | chr11 | NM_000051 | 2.57 |
| D4 | Diffuse | *SDHD* | chr11 | NM_003002 | 2.57 |
| D4 | Diffuse | *ERG* | chr21 | NM_001243428 | 1.42 |
| D4 | Diffuse | *TMPRSS2* | chr21 | NM_005656 | 1.42 |
| D4 | Diffuse | *U2AF1* | chr21 | NM_006758 | 1.42 |
| D1 | Diffuse | *FGFR1* | chr8 | NM_023110 | 2.54 |
| D1 | Diffuse | *NRG1* | chr8 | NM_013964 | 2.54 |
| D1 | Diffuse | *EIF3E* | chr8 | NM_001568 | 2.54 |
| D1 | Diffuse | *EXT1* | chr8 | NM_000127 | 2.54 |
| D1 | Diffuse | *RECQL4* | chr8 | NM_004260 | 2.54 |
| D1 | Diffuse | *RSPO2* | chr8 | NM_178565 | 2.54 |
| D1 | Diffuse | *RET* | chr10 | NM_020975 | 2.88 |
| D1 | Diffuse | *CDH1* | chr16 | NM_004360 | 1.44 |
| D1 | Diffuse | *CTCF* | chr16 | NM_006565 | 1.44 |
| D1 | Diffuse | *CYLD* | chr16 | NM_015247 | 1.44 |
| S3 | Intestinal | *ENO1* | chr1 | NM_001428 | 1.49 |
| S3 | Intestinal | *KIF1B* | chr1 | NM_015074 | 1.49 |
| S3 | Intestinal | *MTOR* | chr1 | NM_004958 | 1.49 |
| S3 | Intestinal | *SDHB* | chr1 | NM_003000 | 1.49 |
| S3 | Intestinal | *AKT3* | chr1 | NM_005465 | 2.51 |
| S3 | Intestinal | *FH* | chr1 | NM_000143 | 2.51 |
| S3 | Intestinal | *PIK3CA* | chr3 | NM_006218 | 2.71 |
| S3 | Intestinal | *SOX2* | chr3 | NM_003106 | 2.71 |
| S3 | Intestinal | *IL7R* | chr5 | NM_002185 | 2.83 |
| S3 | Intestinal | *SKP2* | chr5 | NM_005983 | 2.83 |
| S3 | Intestinal | *TERT* | chr5 | NM_198253 | 2.83 |
| S3 | Intestinal | *PTPRK* | chr6 | NM_001291984 | 2.52 |
| S3 | Intestinal | *ROS1* | chr6 | NM_002944 | 2.66 |
| S3 | Intestinal | *RSPO3* | chr6 | NM_032784 | 2.66 |
| S3 | Intestinal | *ARID1B* | chr6 | NM_017519 | 2.79 |
| S3 | Intestinal | *ESR1* | chr6 | NM_001122742 | 2.79 |
| S3 | Intestinal | *EZR* | chr6 | NM_003379 | 2.79 |
| S3 | Intestinal | *MAP3K4* | chr6 | NM_005922 | 2.79 |
| S3 | Intestinal | *CARD11* | chr7 | NM_001324281 | 2.63 |
| S3 | Intestinal | *EGFR* | chr7 | NM_005228 | 2.63 |
| S3 | Intestinal | *EIF3E* | chr8 | NM_001568 | 2.92 |
| S3 | Intestinal | *RSPO2* | chr8 | NM_178565 | 2.92 |
| S3 | Intestinal | *MYC* | chr8 | NM_002467 | 2.71 |
| S3 | Intestinal | *NFIB* | chr9 | NM_005596 | 2.90 |
| S3 | Intestinal | *RET* | chr10 | NM_020975 | 2.96 |
| S3 | Intestinal | *MAP2K1* | chr15 | NM_002755 | 1.47 |
| S3 | Intestinal | *IGF1R* | chr15 | NM_000875 | 2.63 |
| S3 | Intestinal | *NTRK3* | chr15 | NM_001012338 | 2.63 |
| S3 | Intestinal | *CDH1* | chr16 | NM_004360 | 1.37 |
| S3 | Intestinal | *CTCF* | chr16 | NM_006565 | 1.37 |
| S3 | Intestinal | *ERG* | chr21 | NM_001243428 | 1.49 |
| S3 | Intestinal | *EP300* | chr22 | NM_001429 | 1.49 |
| S3 | Intestinal | *PDGFB* | chr22 | NM_002608 | 1.49 |
| S3 | Intestinal | *RAC2* | chr22 | NM_002872 | 1.49 |
| S4 | Intestinal | *CDC73* | chr1 | NM_024529 | 2.52 |
| S4 | Intestinal | *DDR2* | chr1 | NM_001014796 | 2.52 |
| S4 | Intestinal | *NTRK1* | chr1 | NM_002529 | 2.52 |
| S4 | Intestinal | *SDHC* | chr1 | NM_003001 | 2.52 |
| S4 | Intestinal | *AKT3* | chr1 | NM_005465 | 2.99 |
| S4 | Intestinal | *FH* | chr1 | NM_000143 | 2.99 |
| S4 | Intestinal | *FAT1* | chr4 | NM_005245 | 2.63 |
| S4 | Intestinal | *TERT* | chr5 | NM_198253 | 3.43 |
| S4 | Intestinal | *IL7R* | chr5 | NM_002185 | 2.52 |
| S4 | Intestinal | *SKP2* | chr5 | NM_005983 | 2.52 |
| S4 | Intestinal | *CARD11* | chr7 | NM_001324281 | 2.86 |
| S4 | Intestinal | *PMS2* | chr7 | NM_000535 | 2.58 |
| S4 | Intestinal | *RAC1* | chr7 | NM_006908 | 2.54 |
| S4 | Intestinal | *EGFR* | chr7 | NM_005228 | 3.21 |
| S4 | Intestinal | *BRAF* | chr7 | NM_004333 | 2.60 |
| S4 | Intestinal | *CDK6* | chr7 | NM_001145306 | 2.60 |
| S4 | Intestinal | *KIAA1549* | chr7 | NM_001164665 | 2.60 |
| S4 | Intestinal | *MET* | chr7 | NM_000245 | 2.60 |
| S4 | Intestinal | *MKRN1* | chr7 | NM_013446 | 2.60 |
| S4 | Intestinal | *SMO* | chr7 | NM_005631 | 2.60 |
| S4 | Intestinal | *EZH2* | chr7 | NM_001203247 | 3.12 |
| S4 | Intestinal | *KMT2C* | chr7 | NM_170606 | 3.12 |
| S4 | Intestinal | *FGFR1* | chr8 | NM_023110 | 2.54 |
| S4 | Intestinal | *EIF3E* | chr8 | NM_001568 | 2.54 |
| S4 | Intestinal | *EXT1* | chr8 | NM_000127 | 2.54 |
| S4 | Intestinal | *RECQL4* | chr8 | NM_004260 | 2.54 |
| S4 | Intestinal | *RSPO2* | chr8 | NM_178565 | 2.54 |
| S4 | Intestinal | *POLE* | chr12 | NM_006231 | 2.61 |
| S4 | Intestinal | *CDH1* | chr16 | NM_004360 | 1.45 |
| S4 | Intestinal | *CTCF* | chr16 | NM_006565 | 1.44 |
| S4 | Intestinal | *BRCA1* | chr17 | NM_007294 | 1.39 |
| S4 | Intestinal | *ERBB2* | chr17 | NM_004448 | 1.39 |
| S4 | Intestinal | *NF1* | chr17 | NM_001042492 | 1.39 |
| S4 | Intestinal | *SPOP* | chr17 | NM_001007226 | 1.39 |
| S4 | Intestinal | *STAT3* | chr17 | NM_139276 | 1.39 |
| S4 | Intestinal | *AKT2* | chr19 | NM_001626 | 1.47 |
| S4 | Intestinal | *AXL* | chr19 | NM_021913 | 1.47 |
| S4 | Intestinal | *BAX* | chr19 | NM_138761 | 1.47 |
| S4 | Intestinal | *CIC* | chr19 | NM_015125 | 1.47 |
| S4 | Intestinal | *GNAS* | chr20 | NM_080425 | 2.50 |
| S4 | Intestinal | *SALL4* | chr20 | NM_020436 | 2.50 |
| S4 | Intestinal | *ERG* | chr21 | NM_001243428 | 1.48 |
| S4 | Intestinal | *TMPRSS2* | chr21 | NM_005656 | 1.48 |
| S4 | Intestinal | *U2AF1* | chr21 | NM_006758 | 1.48 |
| S4 | Intestinal | *EP300* | chr22 | NM_001429 | 1.49 |
| S5 | Intestinal | *ENO1* | chr1 | NM_001428 | 1.13 |
| S5 | Intestinal | *KIF1B* | chr1 | NM_015074 | 0.84 |
| S5 | Intestinal | *ARID1A* | chr1 | NM_006015 | 1.06 |
| S5 | Intestinal | *MTOR* | chr1 | NM_004958 | 1.06 |
| S5 | Intestinal | *MUTYH* | chr1 | NM_012222 | 1.26 |
| S5 | Intestinal | *MYCL* | chr1 | NM_001033081 | 1.08 |
| S5 | Intestinal | *SDHB* | chr1 | NM_003000 | 1.06 |
| S5 | Intestinal | *CDKN2C* | chr1 | NM_001262 | 1.31 |
| S5 | Intestinal | *JUN* | chr1 | NM_002228 | 1.31 |
| S5 | Intestinal | *NOTCH2* | chr1 | NM_024408 | 2.61 |
| S5 | Intestinal | *CDC73* | chr1 | NM_024529 | 3.15 |
| S5 | Intestinal | *DDR2* | chr1 | NM_001014796 | 3.15 |
| S5 | Intestinal | *NTRK1* | chr1 | NM_002529 | 3.15 |
| S5 | Intestinal | *SDHC* | chr1 | NM_003001 | 3.15 |
| S5 | Intestinal | *AKT3* | chr1 | NM_005465 | 3.80 |
| S5 | Intestinal | *FH* | chr1 | NM_000143 | 3.80 |
| S5 | Intestinal | *MYCN* | chr2 | NM_001293228 | 3.55 |
| S5 | Intestinal | *ALK* | chr2 | NM_004304 | 3.29 |
| S5 | Intestinal | *EML4* | chr2 | NM_019063 | 3.29 |
| S5 | Intestinal | *EPAS1* | chr2 | NM_001430 | 3.29 |
| S5 | Intestinal | *MSH2* | chr2 | NM_000251 | 3.29 |
| S5 | Intestinal | *MSH6* | chr2 | NM_000179 | 3.29 |
| S5 | Intestinal | *STRN* | chr2 | NM_003162 | 3.29 |
| S5 | Intestinal | *BCL2L11* | chr2 | NM_138621 | 3.02 |
| S5 | Intestinal | *TMEM127* | chr2 | NM_017849 | 3.02 |
| S5 | Intestinal | *NFE2L2* | chr2 | NM_006164 | 3.32 |
| S5 | Intestinal | *SF3B1* | chr2 | NM_012433 | 3.37 |
| S5 | Intestinal | *CASP8* | chr2 | NM_033355 | 2.53 |
| S5 | Intestinal | *CTLA4* | chr2 | NM_005214 | 2.53 |
| S5 | Intestinal | *BARD1* | chr2 | NM_000465 | 3.76 |
| S5 | Intestinal | *ERBB4* | chr2 | NM_005235 | 3.76 |
| S5 | Intestinal | *IDH1* | chr2 | NM_001282387 | 3.76 |
| S5 | Intestinal | *CUL3* | chr2 | NM_003590 | 2.79 |
| S5 | Intestinal | *VHL* | chr3 | NM_000551 | 1.40 |
| S5 | Intestinal | *CTNNB1* | chr3 | NM_001904 | 1.30 |
| S5 | Intestinal | *RHOA* | chr3 | NM_001313941 | 0.96 |
| S5 | Intestinal | *SETD2* | chr3 | NM_014159 | 0.96 |
| S5 | Intestinal | *BAP1* | chr3 | NM_004656 | 1.37 |
| S5 | Intestinal | *PBRM1* | chr3 | NM_018313 | 1.36 |
| S5 | Intestinal | *FGFR3* | chr4 | NM_000142 | 1.40 |
| S5 | Intestinal | *TACC3* | chr4 | NM_006342 | 1.40 |
| S5 | Intestinal | *IL7R* | chr5 | NM_002185 | 3.19 |
| S5 | Intestinal | *SKP2* | chr5 | NM_005983 | 3.20 |
| S5 | Intestinal | *TERT* | chr5 | NM_198253 | 3.20 |
| S5 | Intestinal | *PIK3R1* | chr5 | NM_181523 | 1.33 |
| S5 | Intestinal | *PRDM1* | chr6 | NM_001198 | 3.28 |
| S5 | Intestinal | *ROS1* | chr6 | NM_002944 | 3.89 |
| S5 | Intestinal | *PTPRK* | chr6 | NM_001291984 | 3.00 |
| S5 | Intestinal | *RSPO3* | chr6 | NM_032784 | 3.80 |
| S5 | Intestinal | *EZR* | chr6 | NM_003379 | 2.67 |
| S5 | Intestinal | *MAP3K4* | chr6 | NM_005922 | 3.58 |
| S5 | Intestinal | *EGFR* | chr7 | NM_005228 | 3.64 |
| S5 | Intestinal | *CDK6* | chr7 | NM_001145306 | 3.15 |
| S5 | Intestinal | *MET* | chr7 | NM_000245 | 3.15 |
| S5 | Intestinal | *SMO* | chr7 | NM_005631 | 3.15 |
| S5 | Intestinal | *KIAA1549* | chr7 | NM_001164665 | 3.56 |
| S5 | Intestinal | *EZH2* | chr7 | NM_001203247 | 3.82 |
| S5 | Intestinal | *KMT2C* | chr7 | NM_170606 | 3.82 |
| S5 | Intestinal | *RECQL4* | chr8 | NM_004260 | 1.38 |
| S5 | Intestinal | *FANCC* | chr9 | NM_000136 | 1.22 |
| S5 | Intestinal | *PTCH1* | chr9 | NM_000264 | 1.22 |
| S5 | Intestinal | *ABL1* | chr9 | NM_005157 | 1.06 |
| S5 | Intestinal | *ENG* | chr9 | NM_001114753 | 1.06 |
| S5 | Intestinal | *TSC1* | chr9 | NM_000368 | 1.34 |
| S5 | Intestinal | *NOTCH1* | chr9 | NM_017617 | 1.33 |
| S5 | Intestinal | *RET* | chr10 | NM_020975 | 4.02 |
| S5 | Intestinal | *CCDC6* | chr10 | NM_005436 | 2.74 |
| S5 | Intestinal | *BMPR1A* | chr10 | NM_004329 | 2.70 |
| S5 | Intestinal | *PTEN* | chr10 | NM_000314 | 2.70 |
| S5 | Intestinal | *RRAS2* | chr11 | NM_012250 | 1.26 |
| S5 | Intestinal | *CCND1* | chr11 | NM_053056 | 1.20 |
| S5 | Intestinal | *MEN1* | chr11 | NM_130803 | 1.20 |
| S5 | Intestinal | *CDKN1B* | chr12 | NM_004064 | 1.33 |
| S5 | Intestinal | *ACVR1B* | chr12 | NM_004302 | 1.08 |
| S5 | Intestinal | *ERBB3* | chr12 | NM_001982 | 1.08 |
| S5 | Intestinal | *POLE* | chr12 | NM_006231 | 2.86 |
| S5 | Intestinal | *BRCA2* | chr13 | NM_000059 | 3.99 |
| S5 | Intestinal | *RB1* | chr13 | NM_000321 | 3.01 |
| S5 | Intestinal | *AKT1* | chr14 | NM_005163 | 1.21 |
| S5 | Intestinal | *MAP2K1* | chr15 | NM_002755 | 1.48 |
| S5 | Intestinal | *IGF1R* | chr15 | NM_000875 | 2.93 |
| S5 | Intestinal | *NTRK3* | chr15 | NM_001012338 | 2.93 |
| S5 | Intestinal | *AXIN1* | chr16 | NM_003502 | 1.15 |
| S5 | Intestinal | *CREBBP* | chr16 | NM_004380 | 1.31 |
| S5 | Intestinal | *TRAF7* | chr16 | NM_032271 | 1.15 |
| S5 | Intestinal | *TSC2* | chr16 | NM_000548 | 1.16 |
| S5 | Intestinal | *CDH1* | chr16 | NM_004360 | 1.07 |
| S5 | Intestinal | *CTCF* | chr16 | NM_006565 | 1.07 |
| S5 | Intestinal | *ERBB2* | chr17 | NM_004448 | 1.43 |
| S5 | Intestinal | *NF1* | chr17 | NM_001042492 | 1.43 |
| S5 | Intestinal | *STAT3* | chr17 | NM_139276 | 1.06 |
| S5 | Intestinal | *BRCA1* | chr17 | NM_007294 | 1.05 |
| S5 | Intestinal | *SPOP* | chr17 | NM_001007226 | 1.47 |
| S5 | Intestinal | *PRKAR1A* | chr17 | NM_212471 | 1.40 |
| S5 | Intestinal | *SOX9* | chr17 | NM_000346 | 1.40 |
| S5 | Intestinal | *PPP2R1A* | chr19 | NM_014225 | 2.64 |
| S5 | Intestinal | *GNAS* | chr20 | NM_080425 | 3.43 |
| S5 | Intestinal | *SALL4* | chr20 | NM_020436 | 3.43 |
| S5 | Intestinal | *ERG* | chr21 | NM_001243428 | 1.32 |
| S5 | Intestinal | *TMPRSS2* | chr21 | NM_005656 | 1.19 |
| S5 | Intestinal | *U2AF1* | chr21 | NM_006758 | 1.50 |
| S5 | Intestinal | *CHEK2* | chr22 | NM_007194 | 1.06 |
| S5 | Intestinal | *CRKL* | chr22 | NM_005207 | 1.06 |
| S5 | Intestinal | *MAPK1* | chr22 | NM_002745 | 1.06 |
| S5 | Intestinal | *NF2* | chr22 | NM_000268 | 1.06 |
| S5 | Intestinal | *SMARCB1* | chr22 | NM_003073 | 1.06 |
| S5 | Intestinal | *RAC2* | chr22 | NM_002872 | 1.13 |
| S5 | Intestinal | *EP300* | chr22 | NM_001429 | 0.91 |
| S5 | Intestinal | *PDGFB* | chr22 | NM_002608 | 0.91 |
| D5 | Diffuse | *DPYD* | chr1 | NM_000110 | 2.61 |
| D5 | Diffuse | *NOTCH2* | chr1 | NM_024408 | 2.93 |
| D5 | Diffuse | *DDR2* | chr1 | NM_001014796 | 2.93 |
| D5 | Diffuse | *NTRK1* | chr1 | NM_002529 | 2.93 |
| D5 | Diffuse | *SDHC* | chr1 | NM_003001 | 2.93 |
| D5 | Diffuse | *TPM3* | chr1 | NM_152263 | 2.93 |
| D5 | Diffuse | *BAP1* | chr3 | NM_004656 | 1.42 |
| D5 | Diffuse | *CTNNB1* | chr3 | NM_001904 | 1.41 |
| D5 | Diffuse | *MLH1* | chr3 | NM_000249 | 1.41 |
| D5 | Diffuse | *MYD88* | chr3 | NM_002468 | 1.41 |
| D5 | Diffuse | *PBRM1* | chr3 | NM_018313 | 1.41 |
| D5 | Diffuse | *RAF1* | chr3 | NM_002880 | 1.41 |
| D5 | Diffuse | *RHOA* | chr3 | NM_001313941 | 1.41 |
| D5 | Diffuse | *SETD2* | chr3 | NM_014159 | 1.41 |
| D5 | Diffuse | *VHL* | chr3 | NM_000551 | 1.41 |
| D5 | Diffuse | *PIK3CA* | chr3 | NM_006218 | 2.86 |
| D5 | Diffuse | *PRKCI* | chr3 | NM_002740 | 2.86 |
| D5 | Diffuse | *SOX2* | chr3 | NM_003106 | 2.86 |
| D5 | Diffuse | *TP63* | chr3 | NM_003722 | 2.59 |
| D5 | Diffuse | *TERT* | chr5 | NM_198253 | 3.57 |
| D5 | Diffuse | *IL7R* | chr5 | NM_002185 | 2.68 |
| D5 | Diffuse | *SKP2* | chr5 | NM_005983 | 2.69 |
| D5 | Diffuse | *MAP3K1* | chr5 | NM_005921 | 1.44 |
| D5 | Diffuse | *PIK3R1* | chr5 | NM_181523 | 1.43 |
| D5 | Diffuse | *EGFR* | chr7 | NM_005228 | 2.60 |
| D5 | Diffuse | *CDK6* | chr7 | NM_001145306 | 3.17 |
| D5 | Diffuse | *BRAF* | chr7 | NM_004333 | 2.73 |
| D5 | Diffuse | *KIAA1549* | chr7 | NM_001164665 | 2.73 |
| D5 | Diffuse | *MET* | chr7 | NM_000245 | 2.73 |
| D5 | Diffuse | *MKRN1* | chr7 | NM_013446 | 2.73 |
| D5 | Diffuse | *SMO* | chr7 | NM_005631 | 2.73 |
| D5 | Diffuse | *EIF3E* | chr8 | NM_001568 | 3.30 |
| D5 | Diffuse | *EXT1* | chr8 | NM_000127 | 3.30 |
| D5 | Diffuse | *MYC* | chr8 | NM_002467 | 3.16 |
| D5 | Diffuse | *RECQL4* | chr8 | NM_004260 | 3.30 |
| D5 | Diffuse | *RSPO2* | chr8 | NM_178565 | 3.30 |
| D5 | Diffuse | *CD274* | chr9 | NM_014143 | 1.46 |
| D5 | Diffuse | *CDKN2A* | chr9 | NM_000077 | 1.46 |
| D5 | Diffuse | *JAK2* | chr9 | NM_001322194 | 1.46 |
| D5 | Diffuse | *NFIB* | chr9 | NM_005596 | 1.46 |
| D5 | Diffuse | *TEK* | chr9 | NM_000459 | 1.46 |
| D5 | Diffuse | *GATA3* | chr10 | NM_002051 | 2.87 |
| D5 | Diffuse | *KIF5B* | chr10 | NM_004521 | 2.87 |
| D5 | Diffuse | *RET* | chr10 | NM_020975 | 3.10 |
| D5 | Diffuse | *CCDC6* | chr10 | NM_005436 | 2.50 |
| D5 | Diffuse | *NCOA4* | chr10 | NM_001145262 | 2.50 |
| D5 | Diffuse | *EXT2* | chr11 | NM_207122 | 1.38 |
| D5 | Diffuse | *KRAS* | chr12 | NM_033360 | 3.01 |
| D5 | Diffuse | *ARID2* | chr12 | NM_152641 | 3.41 |
| D5 | Diffuse | *IGF1R* | chr15 | NM_000875 | 1.43 |
| D5 | Diffuse | *SMAD2* | chr18 | NM_001003652 | 1.47 |
| D5 | Diffuse | *SMAD4* | chr18 | NM_005359 | 1.47 |

**Supplementary Table S3. Clinicopathological features of patients in this study**

| Sample No. | Age | Sex | Histology | pT | pN |
| --- | --- | --- | --- | --- | --- |
| D1 | 79 | F | diffuse | pT4a | pN2 |
| D2 | 84 | M | diffuse | pT4a | pN3a |
| D3 | 73 | F | diffuse | pT4a | pN3a |
| D4 | 73 | F | diffuse | pT4a | pN3a |
| D5 | 84 | F | diffuse | pT4a | pN3a |
| S1 | 83 | M | intestinal | pT4a | pN2 |
| S2 | 83 | M | intestinal | pT2 | pN1 |
| S3 | 73 | M | intestinal | pT3 | pN3a |
| S4 | 87 | M | intestinal | pT3 | pN0 |
| S5 | 68 | M | intestinal | pT3 | pN0 |
